# Supplementary material for: Mechanisms of Fibroblast Growth Factor 21 Adsorption on Macroion Layers: Molecular Dynamics Modeling and Kinetic Measurements
Source: Biomolecules. 2023 Nov 26;13(12):1709. doi: 10.3390/biom13121709 (PMC10741725; doi:10.3390/biom13121709)
Supplement: Supplementary file 1 [file biomolecules-13-01709-s001.zip › biomolecules-2713540-supplementary.pdf]

SUPPORTING INFORMATION

Mechanism of Fibroblast Growth Factor 21 Adsorption on Macroion Layers: Molecular Dynamics Modeling and Kinetic Measurements

Monika Wasilewska[1], Maria Dąbkowska[2], Agata Pomorska[1], Piotr Batys[1], Bogusław Kowalski[2], Aneta Michna[1*], Zbigniew Adamczyk[1*]

[1] *Jerzy Haber Institute of Catalysis and Surface Chemistry, Polish Academy of Sciences, Niezapominajek 8, PL-30239 Krakow, Poland*

[2] *Independent Laboratory of Pharmacokinetic and Clinical Pharmacy, Pomeranian Medical University, Rybacka 1, 70-204 Szczecin, Poland*

*Corresponding author:

Zbigniew Adamczyk
Jerzy Haber Institute of Catalysis and Surface Chemistry, Polish Academy of Sciences, Niezapominajek 8, PL-30239 Krakow, Poland
e-mail: zbigniew.adamczyk@ikifp.edu.pl

Aneta Michna
Jerzy Haber Institute of Catalysis and Surface Chemistry, Polish Academy of Sciences, Niezapominajek 8, PL-30239 Krakow, Poland
e-mail: aneta.michna@ikifp.edu.pl

1. Hydrodynamic diameters of FGF 21 (peak intensities, MADLS)

The analyses performed on peak intensity showed the presence of the three peaks (see Fig. S1). FGF 21 maintained self-assembled protein molecules with hydrodynamic diameters ranging from 5.5 nm to 4.6 nm for 0.01 M and 0.15 M, respectively (first peak by intensity) to 440 nm (third peak by intensity). These peaks appear to be relatively stable over time.

a)

b)

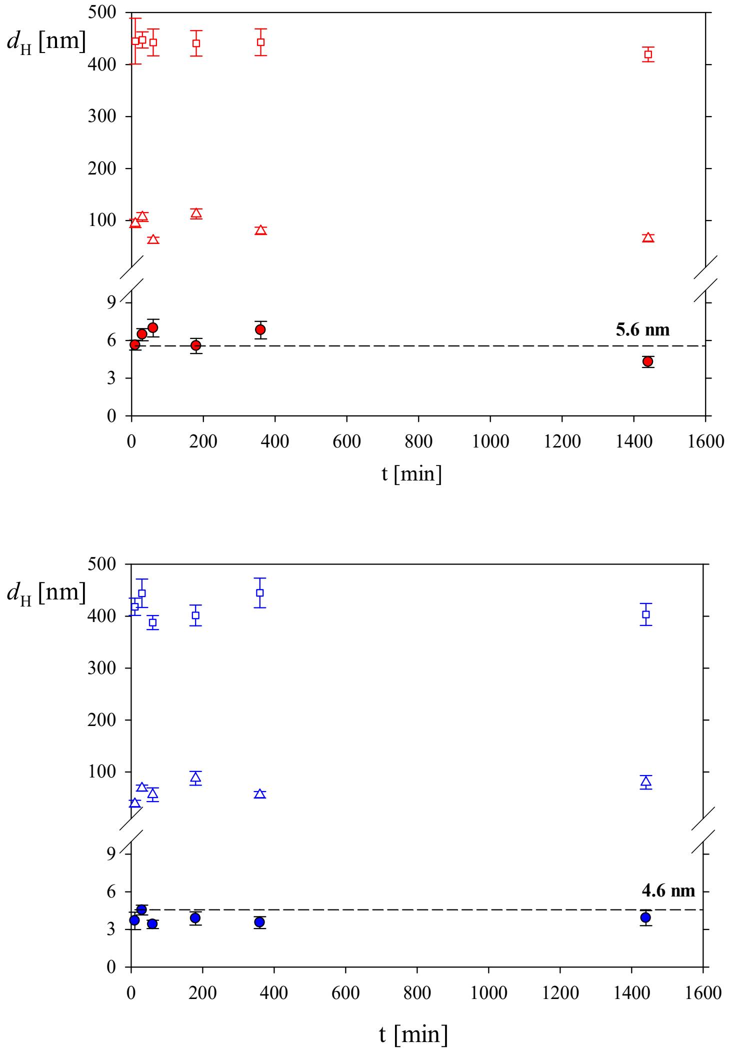

Fig. S1. The dependences of the hydrodynamic diameter (by peak intensity) (d_H) on time determined at pH 7.4 and ionic strength of a) 0.01 M and b) 0.15 M.

As can be seen in Fig. S1, the hydrodynamic diameters (determined as the lowest peaks by intensity for the ionic strengths of 0.01 and 0.15 M NaCl) are 5.6 and 4.6 nm. They are slightly higher compared to the peak numbers (5.4 and 3.7 nm, for the same ionic strength range) presented in the main manuscript. The second and third peaks evaluated by intensity

are also present, with values much higher than those presented as the first peaks. It should be noticed, however, that large particles scatter much more light, and even a small number of large particles can cause the contribution to smaller particles [1].

2. CHO-K1 cell viability

Having established the stability of the PDADMAC/FGF 21 layer in vitro, the authors proceeded to investigate basic toxicity by examining the constituents of the layer, specifically PDADMAC and FGF 21, as well as a combination denoted as PDADMAC/FGF 21 (see Figure S2). This assessment was conducted using two noncancerous cell lines, CHO-K1 and L-929 (results were presented in the main manuscript), which are commonly employed in industrial biotechnology and toxicology research. As a control, cells were not exposed to FGF 21, PDADMAC, or the combination of both FGF 21 and PDADMAC.

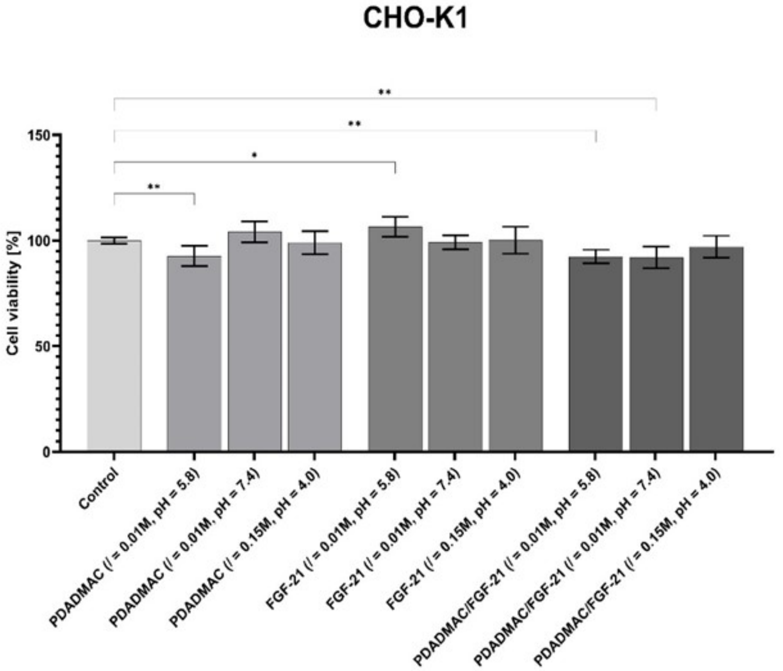

Figure S2. The influence of PDADMAC, FGF 21, and PDADMAC/ FGF 21 layer on viability (in relation to untreated control) of CHO-K1 cells. They were treated with components in different adsorption conditions (described in the picture as 0.01 M NaCl or 0.15M NaCl, pH = 4.0, 5.8, 7.4) and kept in regular cell culture conditions. After 24 hours AlamarBlue™ reagent was added to cell culture according to the manufacturer's instruction. The experiment was performed in three independent replicates. One-way ANOVA and

Dunnett's multiple comparisons test statistical analysis (GraphPad Prism) shows significant differences.

Similarly to the results presented in the main manuscript, FGF 21 caused no toxicity in CHO-K1 cells after 24 hours of incubation. When the cells were cultured on the PDADMAC layer (0.01M NaCl pH 5.8), a significant decrease in viability was also observed for CHO-K1 cells (88.9% ± 2.9%) (Figure S2).

The adsorption of FGF 21 onto the PDADMAC layer from 0.01 M NaCl at pH 5.8/7.4 also reduced the viability of CHO-K1 to 92.4% ± 5.3%. Neither PDADMAC nor the PDADMAC/FGF 21 layers, obtained from 0.15 M NaCl, pH 4.0, led to any significant deterioration in studied cell lines as was shown in the tests using the AlamarBlue assay.

Naumenko et al. conducted a study on various cationic macroions, including PDADMAC, and found increased cytotoxicity, with an IC50 of 20-25 μg per 10^5 cells [2]. Nonetheless, we could not compare both studies directly because of different macroion concentrations, the number of cells, and the in vitro formation of the PDADMAC layer. It was shown that the cytotoxicity of PDADMAC seems to depend on the composition of layers and cell type. Moreover, certain publications report a positive effect of PDADMAC-based macroion multilayers, [3], [4], which improve fibroblasts attachment, viability and spreading. On the contrary, in a study focused on muscle cells, we discovered that the PDADMAC-terminated layer increased cell death, indicating that it was not a compatible surface for these cells [5].

REFERENCES

1. Mourdikoudis, S.; Pallares, R.M.; Thanh, N.T.K. Characterization Techniques for Nanoparticles: Comparison and Complementarity upon Studying Nanoparticle Properties. *Nanoscale* **2018**, *10*, 12871–12934, doi:10.1039/c8nr02278j.
2. Naumenko, E.; Akhatova, F.; Rozhina, E.; Fakhrullin, R. Revisiting the Cytotoxicity of Cationic Polyelectrolytes as a Principal Component in Layer-by-Layer Assembly Fabrication. *Pharmaceutics* **2021**, *13*, 1230, doi:10.3390/pharmaceutics13081230.
3. Dubas, S.T.; Kittitheeranun, P.; Rangkupan, R.; Sanchavanakit, N.; Potiyaraj, P. Coating of Polyelectrolyte Multilayer Thin Films on Nanofibrous Scaffolds to Improve Cell Adhesion. *J. Appl. Polym. Sci.* **2009**, *114*, 1574–1579, doi:10.1002/app.30690.
4. Kidambi, S.; Lee, I.; Chan, C. Controlling Primary Hepatocyte Adhesion and Spreading on Protein-Free Polyelectrolyte Multilayer Films. *J. Am. Chem. Soc.* **2004**, *126*, 16286–16287, doi:10.1021/ja046188u.
5. Muzzio, N.E.; Pasquale, M.A.; Gregurec, D.; Diamanti, E.; Kosutic, M.; Azzaroni, O.; Moya, S.E. Polyelectrolytes Multilayers to Modulate Cell Adhesion: A Study of the Influence of Film Composition and Polyelectrolyte Interdigitation on the Adhesion of the A549 Cell Line. *Macromol. Biosci.* **2016**, *16*, 482–495, doi:10.1002/mabi.201500275.
